# Considerations on the calculation of fractions of cardiovascular disease attributable to psychosocial work factors: Comment on: Niedhammer I, Sultan-Taïeb H, Chastang JF, Vermeylen G, Parent-Thirion A. Fractions of cardiovascular diseases and mental disorders attributable to psychosocial work factors in 31 countries in Europe

**DOI:** 10.1007/s00420-013-0919-0

**Published:** 2013-11-28

**Authors:** E. Backé, H. Burr, U. Latza

**Affiliations:** Work and Health, Federal Institute for Occupational Safety and Health, Noeldnerstraße 40-42, 10317 Berlin, Germany

Dear Editor,

Motivated by the recent publication of Niedhammer et al. (2013) we would like to communicate some in our view noteworthy considerations concerning the measurement of psychosocial stress in epidemiological studies and the calculation of the population attributable fraction based on these studies with regard to research aimed at the prevention of disease.

Changes in the workplace and in the working population lead to a continuous steep increase in the literature on the association of psychosocial stress experienced at the workplace and disease in particular cardiovascular diseases (CVD) (reviewed by Kivimäki et al. 2006, 2012; Backé et al. 2012; Eller et al. 2009; Belkic et al. 2004). Also in the recent publication of Niedhammer et al. (2013), population attributable fractions (PAF) for psychosocial work factors were calculated in relation to CVD and mental diseases. The choice of the concept of the PAF is reasonable in order to translate epidemiological evidence into policy and practice in the field of cardiovascular health in the workplace. The proportion of cases (morbidity and mortality) in a population attributable to a given exposure should provide information on most urgent factors that need to be addressed in prevention strategies.

Most of the studies on CVD investigate the association between job strain measured by the Job Content Questionnaire (JCQ) (Karasek et al. 1998). Fewer studies use the effort–reward imbalance (ERI) model (Siegrist et al. 2004) or the organisational injustice model (Elovainio et al. 2006) or other instruments. There are different ways to derive PAFs for a population (e.g., country or region), either directly from a population-based study or indirectly. With the indirect approach, risk estimates from one or more analytical studies are retrieved and combined with information on the fraction of exposed persons in the general population from other sources (mainly surveys). Risk estimates may be derived from studies selected based on specific quality criteria (e.g., a certain design and/or statistical model including the relevant confounders) or from meta-analyses, respectively. When using this method, survey questions to estimate the prevalence of exposure need to be comparable to the instruments used for the exposure in the observational studies, which are the basis for the calculation of risk estimates. Validity of the PAF depends heavily on the estimation of the prevalence as well as risk estimates, given that they are correctly estimated (Olsen 1995). Niedhammer et al. (2013) used proxies for the job strain and effort–reward imbalance from the fourth European Working Condition Survey (EWCS) and combined the prevalences with risk estimates from published meta-analyses. With this indirect method, the authors describe PAFs between 2.51 and 5.77 % for job strain and 9.78–27.89 % for the effort–reward ratio >1 in the European countries.

Reviewing the literature on fractions of CVD attributable to psychosocial work factors, we also saw that the estimated PAFs differ severely between countries (Backé et al. 2013; Backé and Latza 2013). With the indirect approach, PAFs for cardiovascular outcomes attributed to occupational stress have been derived for the United States (Steenland et al. 2003), Finland (Nurminen and Karjalainen 2001), Korea (Ha et al. 2011), and France (Sultan-Taïeb et al. 2011). For Sweden, PAFs in relation to several diseases were calculated by Järvholm et al. (2013). Here, with respect to job strain and myocardial infarction, calculations with the direct approach were based on a population-based case reference study (Peter et al. 2002). Illustrated for those European countries, where information about PAFs (besides the calculations based on EWCS) are available, PAF estimates differ depending on different prevalence of the exposure but also on different choices in the selection of studies indicating the risk estimates (Table 1). Besides, also discussed by Niedhammer et al. (2013), some authors choose age- and gender-adjusted risk estimates, and some multiple-adjusted risk estimates, respectively. The latter may result in an underestimation of the relative risk when mediators such as high blood pressure or high cholesterol are included. In a recent meta-analysis (Kivimäki et al. 2012) that also included hitherto unpublished data, the overall PAF for job strain related to CVD in Europe is denominated with 3.4 %.

**Table 1:** Population attributable fractions [PAF%, 95 % confidence intervals (CI), if available] for occupational stress related to cardiovascular diseases in different countries estimated with different methods

	Germany	Finland^a^	Sweden^b^	France^c^	Europe
Job strain		M 16 % F 19 %	M 6.7 % F 14.7 %	6.5–25.5 %	3.40 %^d^ (CI 1.5–5.4)
Proxy EWCS^e^	5.23 % (CI 1.49–8.97)	3.85 % (CI 1.06–6.64)	2.86 % (CI 0.75–4.96)	3.65 % (CI 1.00–6.31)	4.46 % (CI 1.26–7.65)
ERI	1.2–25.7 %^f^				
Proxy EWCS^e^	19.5 % (CI −2.51 to 40.82)	17.16 % (CI −2.71 to 37.03)	16.44 % (CI −2.75–35.64)	18.83 % (CI 2.45–40.19)	18.21 % (CI 2.58–39.01)

*EWCS* European Working Conditions Survey

^a^Nurminen and Karjalainen (2001), *m* males, *f* females, PAF for shift work, involving work strain

^b^Järvholm et al. (2013), *m* males, *f* females

^c^Sultan-Taïeb et al. (2011)

^d^Kivimäki et al. (2012)

^e^Niedhammer et al. (2013)

^f^Backé et al. (2013)

Apart from the differences in methods to estimate the prevalence of job strain (e.g., complete questionnaire or proxy measures) as well as the selection of studies giving information on risk estimates for the association of CVD and job strain, there is another issue that needs to be addressed. Within the Karasek model, job strain is defined by the presence of high demand combined with low decision latitude. Median cut points are used to define high demand, low control, and job strain. This is arbitrary. Further cutoffs vary depending on the structure of occupations within the population. If one supposes that levels of demand and control differ between countries (Moncada et al. 2010) and given the lack of a population-independent cutoff for job stress, identical answers to the demand and control scales may be considered as low stress in one country and as high stress in another country. This point is also mentioned by Niedhammer et al. as possible limitation of their study. But additionally the question remains whether these frequencies calculated within the Karasek model are comparable to other psychosocial job exposure prevalence rates that can theoretically reach 100 % (e.g., the number of subjects working more than 48 h a week). Job strain by definition is one of four categories in the model, resulting from dichotomization of the demand scale and the control scale that can maximally reach 50 %.

Also for the estimation of PAFs for ERI, some methodological problems need to be discussed: the risk estimates used to calculate PAFs are based on studies comparing high effort–reward imbalance (upper tertile or quartile) with the baseline quantile (Kuper et al. 2002; Kivimäki et al. 2002). It is questionable whether risk estimates for upper quantiles can be combined with prevalence estimates for effort–reward imbalance above 1 obtained from surveys.

Taking all available evidence together, assuming a causal relationship, we now know that the PAFs for occupational stress defined by job strain or effort–reward imbalance related to CVD in European countries may be in a range between 3 and 25 % (with wide confidence intervals) (see Table 1). But how do we translate this information into prevention strategies?

Models for the description of occupational stress are valuable because they combine many psychosocial issues. However, besides difficulties to obtain reliable prevalence data, e.g., on job strain, the investigation of defined single psychosocial factors or other (forthcoming) dimensions of psychosocial exposures at the workplace is not included in the models. Since effective interventions to reduce stress at the workplace need to be targeted to preventable risk factors, new data will be necessary and helpful. Well-defined psychosocial work factors measured by valid instruments need to be included into the National surveys. These factors as well as novel factors have to be investigated prospectively with respect to disease in cohort studies, which should include repeated measurements of the “stressful” exposure. With this information, more specific PAFs can be calculated to prioritize the most important psychosocial issues in prevention policies at the workplace. This is, as also addressed by Niedhammer et al. (2013), important not only in the context of CVD but also in the context of other diseases such as depression.
